# Diagnosing X-Linked Adrenoleukodystrophy after Implementation of Newborn Screening: A Reference Laboratory Perspective

**DOI:** 10.3390/ijns9040064

**Published:** 2023-11-02

**Authors:** Julia Prinzi, Marzia Pasquali, Judith A. Hobert, Rachel Palmquist, Kristen N. Wong, Stephanie Francis, Irene De Biase

**Affiliations:** 1 Department of Human Genetics, Graduate Program in Genetic Counseling, University of Utah, Salt Lake City, UT 84112, USA; 2 Department of Pathology, University of Utah School of Medicine, Salt Lake City, UT 84112, USA; 3 ARUP Laboratories, Salt Lake City, UT 84108, USA; 4 Department of Pediatrics, University of Utah School of Medicine, Salt Lake City, UT 84112, USAkristen.wong@hsc.utah.edu (K.N.W.)

**Keywords:** adrenoleukodystrophy, very long-chain fatty acids, newborn screening, Zellweger spectrum disorders, pre-symptomatic disease detection

## Abstract

Adrenoleukodystrophy (ALD) is caused by pathogenic variants in the *ABCD1* gene, encoding for the adrenoleukodystrophy protein (ALDP), leading to defective peroxisomal β-oxidation of very long-chain and branched-chain fatty acids (VLCFA). ALD manifests in both sexes with a spectrum of phenotypes, but approximately 35% of affected males develop childhood cerebral adrenoleukodystrophy (CCALD), which is lethal without hematopoietic stem cell transplant performed before symptoms start. Hence, ALD was added to the Recommended Uniform Screening Panel after the successful implementation in New York State (2013–2016). To date, thirty-five states have implemented newborn screening (NBS) for ALD, and a few programs have reported on the successes and challenges experienced. However, the overall impact of NBS on early detection of ALD has yet to be fully determined. Here, we conducted a retrospective analysis of VLCFA testing performed by our reference laboratory (ARUP Laboratories, Salt Lake City, UT, USA) over 10 years. Rate of detection, age at diagnosis, and male-to-female ratio were evaluated in patients with abnormal results before and after NBS implementation. After NBS inclusion, a significant increase in abnormal results was observed (471/6930, 6.8% vs. 384/11,670, 3.3%; *p* < 0.0001). Patients with ALDP deficiency identified via NBS were significantly younger (median age: 30 days vs. 21 years; *p* < 0.0001), and males and females were equally represented. ALD inclusion in NBS programs has increased pre-symptomatic detection of this disease, which is critical in preventing adrenal crisis as well as the severe cerebral form.

## 1. Introduction

Adrenoleukodystrophy (ALD; MIM: 300100) is caused by pathogenic variants in the ATP binding cassette subfamily D member 1 (*ABCD1*) gene [1,2]. *ABCD1* resides on the X-chromosome and encodes for the adrenoleukodystrophy protein (ALDP), a peroxisomal transmembrane protein required for transport of very long-chain fatty acids (VLCFA) into peroxisomes, where they undergo β-oxidation [1,3,4]. Defects in ALDP impair VLCFA uptake into peroxisomes, leading to VLCFA accumulation in body fluids and tissues, especially in the nervous system and adrenal glands [5,6,7]. ALDP deficiency is the most common peroxisomal disorder, with an estimated incidence of ~1 in 17,000 births [8,9]. Even higher incidences have been reported via newborn screening (NBS) programs: ~1 in 10,500 births in aggregate [9,10,11,12,13,14,15]. This difference may reflect identification via NBS of mild forms of disease previously unrecognized or the detection of individuals that will never develop disease. Moreover, NBS increases the detection of variants of uncertain significance (VUS) that can cause significant diagnostic uncertainty. However, we anticipate that long-term, longitudinal follow-up of infants with abnormal screens will ultimately lead to a better understanding of this disease.

Both males and females can be affected with ALDP deficiency, and the clinical manifestation is highly variable. Neither age of onset nor severity can be predicted; no genotype-phenotype correlation has been documented, and low concordance rates among twin/non-twin sibling pairs have been observed [4,16]. It is rare, however, for males to remain asymptomatic past 40–50 years of life [4,17,18], whereas approximately one third of females never develop symptoms, possibly a result of the underlying X-inactivation pattern.

ALDP deficiency manifests primarily with three clinical phenotypes: (a) isolated primary adrenal insufficiency (PAI), (b) myelopathy with or without peripheral neuropathy (adrenomyeloneuropathy, AMN), and (c) progressive inflammatory white matter demyelination cerebral form, which includes childhood cerebral ALD (CCALD) and adolescent cerebral ALD (AdolCALD) [19]. PAI is common in males, with ~80% of patients developing symptoms [20]; however, it is rare in females, affecting 1% or less of women carrying pathogenic variants in *ABCD1* [17,21]. AMN is the most common of the three clinical presentations, manifesting as a slowly progressive spastic paraparesis and sensory ataxia, with onset between the second and fifth decade of life in men and in the fifth decade in women [4,18,22,23]. Currently, treatment options for spinal cord disease are mainly supportive, with new curative therapies currently under development [24]. Approximately 20% of patients with AMN experience rapid development of severe cognitive and motor disabilities, with death occurring within 5 to 10 years of symptom onset [4,18,22].

Finally, the cerebral form is associated with progressive inflammatory white matter demyelination and most often presents in childhood (CCALD) but can also present later in adolescence (AdolCALD), or more rarely in adulthood. Approximately 35% of males with ALDP deficiency develop CCALD, while cerebral ALD is exceedingly rare in females [4,22,23]. White matter lesions visible through MRI precede symptoms and are the first signs used to detect cerebral disease [4]. Typical clinical presentation for CCALD includes cognitive deficits and behavioral problems that are commonly attributed to other conditions, such as attention deficit hyperactivity disorder (ADHD). CCALD is lethal without hematopoietic stem cell transplant. This transplant can provide long-term stabilization and occasionally reverse early-stage cerebral involvement [3,25]. However, it is only clinically effective in reversing neurocognitive impairment when performed at an early stage of cerebral demyelination, underscoring the need for early detection of ALD.

Hence, on 30 December 2013, New York State initiated NBS for ALD to facilitate early detection. Their successful program ran from 2013 to 2016 and provided the foundation for inclusion of ALD in the Recommended Uniform Screening Panel (RUSP) by the US Secretary of the Department of Health and Human Services [26,27]. As of January 2023, thirty-five of the United States have implemented NBS for ALD (Table 1). Although designed to identify males affected with ALD, NBS has also identified female carriers of this condition, who can develop symptoms later in life, as well as other peroxisomal disorders that impair VLCFA β-oxidation, including peroxisome biogenesis defects (PBD) of the Zellweger spectrum (Zellweger spectrum disorders, ZSD) [7]. ZSD are caused by deficiency of any of the 13 Peroxisomal Biogenesis Factor (PEX) proteins required for peroxisome biogenesis, and ZSD clinically present with liver dysfunction, neurological abnormalities, developmental delays, adrenocortical dysfunction, and vision and hearing impairment [28]. Diagnostic confirmation for the abnormal screens and the identification of the specific defect requires a combination of biochemical testing, including quantitation of VLCFA and branched-chain fatty acids in plasma, and molecular genetic testing.

In this study, we aimed to assess the impact of inclusion of ALD in NBS on the detection of ALD and other peroxisomal disorders through a retrospective analysis of VLCFA testing performed by our reference laboratory (ARUP Laboratories, Salt Lake City, UT, USA) over a 10-year period. Age at diagnosis and sex distribution of patients with ALDP deficiency were evaluated before and after NBS implementation.

## 2. Methods

This study was reviewed and approved by the Institutional Review Board at the University of Utah.

### 2.1. Laboratory Studies

VLCFA and branched-chain fatty acid testing was performed according to standard procedures [29]. Briefly, samples undergo acid hydrolysis to free the fatty acids from their coenzyme A esters; the fatty acids are subsequently derivatized using oxalyl chloride, dimethylaminoethanol, and finally methyl iodide. The trimethyl-amino-ethyl iodide ester derivatives are analyzed using liquid chromatography-tandem mass spectrometry (LC-MS/MS) in positive electrospray ionization and selective reaction monitoring mode. This method simultaneously quantifies very long-chain fatty acids (C22:0–C26:0) and branched-chain fatty acids (pristanic acid and phytanic acid). The ratios C26:0/C22:0, C24:0/C22:0, and pristanic/phytanic acid are also calculated. 

### 2.2. Study Population

The data compiled for this study derive from specimens submitted from 48 of the United States of America and encompasses all VLCFA and branched-chain fatty acid results reported by our laboratory between 1 January 2012 and 30 July 2022. This period included two years preceding the inclusion of Adrenoleukodystrophy (ALD) in the New York NBS program (start date 30 December 2013). Since NBS is managed at the state level, the testing start dates for inclusion of ALD in NBS protocols differs among the states. Therefore, to identify the state-specific dates (day/month/year or month/year) for ALD inclusion into NBS programs we used available information from NBS program web sites, press releases, and the published literature (Table 1). These dates were used on a state-by-state basis to discriminate between samples submitted prior to implementation of ALD NBS or afterwards. This data was then used to analyze detection rates before and after NBS inclusion.

VLCFA and branched-chain fatty acid results for each patient were evaluated at time of sample submission in comparison to normal reference intervals. All patient results were retrospectively compiled and grouped by interpretation as: normal, suggestive of dietary artifacts and/or liver dysfunction, or consistent with a peroxisomal disorder. In the majority of cases with an abnormal result consistent with a peroxisomal disorder diagnosis, the reason for referral and the suspected or confirmed diagnosis (ALDP deficiency, ZSD or other peroxisomal disorders) as well as further patient information (phenotype description, family history) were collected at the time of reporting the testing results from the referring physician or the send-out laboratory. Information was either extracted from a history form, submitted with the sample, or obtained by an ARUP genetic counselor by directly contacting the provider (typically by phone). In few cases, we recontacted the providers for further information during this study. A number of providers could not be reached after multiple attempts, or the information obtained was limited. When multiple VLCFA tests were submitted for the same patient, only the first result was categorized as “diagnostic”. All the available patient information (demographic data, phenotype description, family history) together with VLCFA results and results from other laboratory tests, including molecular genetic testing, were used for this study.

### 2.3. Statistics

Medians and range (minimum to maximum value) are presented in Table 2. Two-sided Mann–Whitney test was used to perform comparisons between medians, after confirming non-Gaussian distribution. Comparison of sex distribution between groups was performed using the chi-squared test. Results were considered statistically significant with two-tailed *p* value < 0.05. The Kruskal–Wallis test was used to test differences in age distribution between groups, after confirming non-Gaussian distribution; adjusted *p* value < 0.05 were considered significant. GraphPad Prism^®^ software [Version 9.2.0 (2021); Dotmatics, Boston, MA, USA] and Microsoft Excel (2016; Microsoft Corporation, Redmond, WA, USA) were used for data analysis.

## 3. Results

A total of 18,600 results for plasma very long-chain and branched-chain fatty acid (VLCFA) testing were reported by our laboratory between 1 January 2012 and 30 July 2022. Of these results, 10,255 were from males, 8272 from females, and 73 from individuals where sex was not specified or was indicated as ‘unknown’. Age at collection ranged from day of birth to 96 years, with 48% of patients less than 12 years of age and 19% less than one year of age. The wide range of patient ages at evaluation likely reflects the broad differential diagnosis for this group of clinically heterogenous disorders. Most samples were reported as normal (81.4%; 15,136/18,600) or suggestive of dietary artifacts and/or liver dysfunction (14.0%; 2609/18,600). Only 4.6% of the samples submitted (855 samples; 577 from males, 278 from females) had results consistent with a peroxisomal disorder, a percentage consistent with previous reports [6]. Overall, the patients with abnormal results were younger (71% below 18 years of age vs. 55%) and predominantly male (67% vs. 56%) compared to all samples submitted. 

**Figure 1:**
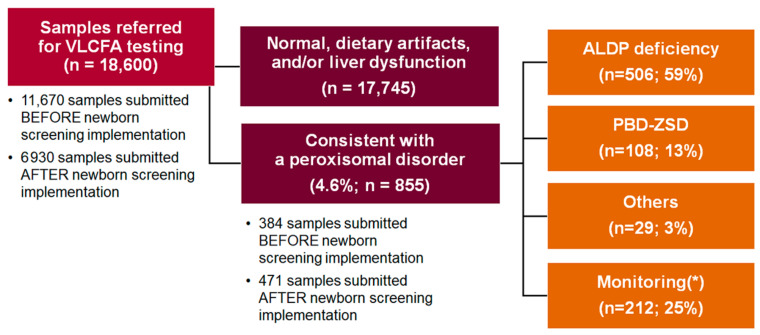
A visual summary of VLCFA testing results reported by our reference laboratory between 1 January 2012 and 30 July 2022. PBD-ZSD = peroxisome biogenesis defects-Zellweger spectrum disorders; PD = peroxisomal disorders; (*) samples sent to monitor VLCFA levels in known patients.

As previously mentioned, our cohort included samples collected both before and after states included ALD in their NBS protocol. Table 1 documents the status of Adrenoleukodystrophy newborn screening by state, as of January 2023. Using this information, we were able to determine that 11,670 of the samples received were submitted prior to NBS for ALD and 6930 samples were submitted after NBS had been implemented. After the inclusion of ALD in the NBS protocol, the rate of abnormal results doubled (471/6930, 6.8% vs. 384/11,670, 3.3%; *p* < 0.0001). Both before and after inclusion, a comparable number of patients were referred because of clinical findings; however, after inclusion, additional patients were referred because of an abnormal screen. Based on patient age and reason for referral, this observed increase was not exclusively due to diagnosing newborns with a positive newborn screen but also included older family members of infants identified through an abnormal NBS. As expected, the majority of abnormal VLCFA results (643/855, 75%) were diagnostic samples, with ALDP deficiency (males or females; 506/855, 59%) representing the most common diagnosis (Figure 1). The remaining 25% of abnormal results (*n* = 212) were ordered to monitor VLCFA levels in known patients (on average, 2.8 results for patient), often to evaluate therapy (e.g., phytanic acid levels in Refsum disease). 

Among the 643 diagnostic samples, a specific diagnosis was confirmed by the provider for 238 cases, and abnormal molecular genetic test results were reported for the proband or a family member in 117 cases. Patient clinical status was provided in 458/643 cases, including 147 patients reported as asymptomatic, although clinical findings were not always described in detail. A positive family history was reported in 147/643 cases. In some patients, VLCFA results were specific to a peroxisomal disorder (e.g., markedly elevated phytanic acid in Refsum disease); however, increases in VLCFAs, and particularly C26:0 and the C26:0/C22:0 ratio, were detected in both ALDP deficiency and ZSD, as previously described [6,28]. Nonetheless, significant differences were detected between the ZSD and ALDP groups, with higher values observed in ZSD (e.g., median C26:0 = 4.4 vs. 1.6 mmol/L, and median C26:0/C22:0 = 0.20 vs. 0.03, in ZSD and ALDP deficiency, respectively; *p* value < 0.0001). Taken together, all available information, the results from plasma VLCFA testing, and other available test results allowed for a high degree of confidence in the diagnosis in most diagnostic cases (532/643). 

We had enough clinical phenotype information to categorize 141 ALDP deficiency cases into three main phenotypes, i.e., isolated PAI, AMN, or cerebral ALD (Table 2). PAI was reported in 21% of symptomatic patients (30/141), one of which was female. In 23 out of 30 patients, PAI was the only reported clinical finding. Adrenal insufficiency was present concurrently with the AMN or cerebral ALD phenotype in an additional five and two patients, respectively. AMN was the most common phenotype described in our cohort (80/141; 57%), and it was reported in both sexes (50 males, 30 females); three patients with AMN also had symptoms of progression to cerebral ALD (all males; ages 36 to 53 years). Cerebral ALD was reported in 27% of the symptomatic patients (38/141), all males, with the majority being 6 to 12-year-old boys. Abnormally elevated VLCFA consistent with ZSD accounted for 13% of the abnormal results. Only five of these patients for whom phenotype information was available (*n* = 94) were asymptomatic at the time of diagnosis; most ZSD patients presented early with a severe multi-system involvement. 

**Figure 2:**
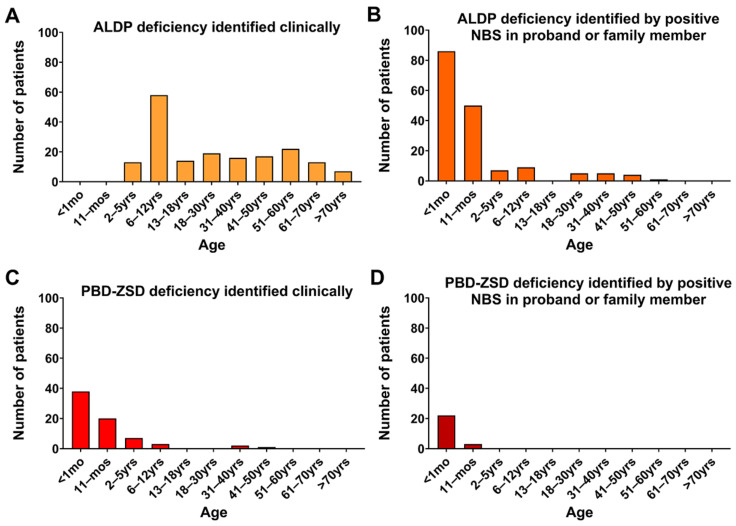
Age distribution of patients with ALDP deficiency (**A**,**B**) or PBD-ZSD (**C**,**D**) based on reason for referral.

In the majority of the ALDP deficiency and ZSD cases (504/614), there was enough information to determine the reason for sample referral (Table 3). Most patients with ALDP deficiency referred because of clinical findings were males (145/179, *p* value < 0.0001), while males (70/137) and females (67/137) were equally represented in patients identified because of a positive newborn screen. A statistically significant difference between the number of males and females was not present in ZSD patients (*p* value =0.439; Table 3). Similar findings were obtained when only results with a high degree of confidence were included. Relative to the reason for referral, a statistically significant difference was also seen in the age at diagnosis in patients with ALDP deficiency. Specifically, patients evaluated because of clinical findings were older than patients identified because of an abnormal NBS in the proband or a family member (adjusted *p* value < 0.0001; Figure 2A,B), with 31% (56/179) being 6 to 12-year-old boys when the clinical diagnosis was made. Most females presented clinically as adults (>18 years); there were no patients diagnosed clinically before one year of age. A positive family history was the indication in 58 cases; these patients were also significantly older than those with a positive NBS (adjusted *p* value < 0.0001), but no significant difference was noted with patients presenting clinically (adjusted *p* value > 0.9999). In contrast, a positive NBS did not significatively decrease age of diagnosis for ZSD patients (adjusted *p* value > 0.9999; Figure 2C,D). Indeed, 84% of our results diagnostic for ZSD were obtained in patients less than a year old regardless of indication for testing, and most ZSD patients referred for abnormal NBS were already symptomatic at the time of follow-up.

## 4. Discussion

As of January 2023, thirty-five states have successfully added ALD to the conditions screened via NBS. Several state programs [10,12,13,14,15] have described their experience, generally confirming the data first published after the start of the New York State program (2013–2016) [9]. ALDP deficiency is a relatively common disorder (~1 in 10,500 births in aggregate), and infants are reliably identified by an increased C26:0 lysophosphatidylcholine (C26:0-LPC) in dried blood spots. The false-positive rate associated with this biomarker is very low; however, other peroxisomal disorders without effective treatment can also present elevated C26:0-LPC, since this biochemical abnormality is not specific to ALDP deficiency. C26:0-LPC has shown similar sensitivity in detecting female infants carrying *ABCD1* pathogenic variants, who may eventually develop symptoms in adulthood, compared to males. Moreover, a positive screen has implications for the affected infant’s family members, potentially prompting a diagnosis of ALDP deficiency in their mother, siblings, or other maternal relatives, without the ability to predict disease severity or age of onset. Lastly, ALD symptoms may never materialize in infants identified via NBS, even when biochemical evidence of impaired VLCFA metabolism is confirmed, considerably increasing the difficulties of interpreting variants of uncertain significance. 

In spite of the challenges and ethical issues [30], NBS for ALD has been successful at detecting CCALD before symptoms develop, preventing the inflammatory white matter demyelination [31]. Most children with ALD detected clinically or through NBS did not have a positive family history, as seen in this study and in the literature [14]. Without a family history and/or asymptomatic screening, CCALD is typically diagnosed when irreversible damage is already present, strongly supporting inclusion on NBS panels. However, the overall impact of NBS on early detection of ALD has yet to be fully appreciated, in part because of the slow adoption by NBS programs [27]. Our study attempted to evaluate the difference made by NBS on ALDP deficiency detection rate, age at diagnosis, and male to female ratio. Unlike data published by NBS programs, our cohort is representative of diagnostic testing performed in a large reference laboratory, and thus it captures patients seen for a broad variety of reasons, including follow-up of NBS. Few large retrospective analyses of VLCFA data have been published; however, those studies have focused on analyte levels observed in these patients, which is outside the scope of this study [6,32].

A limitation of our retrospective analysis is the lack of clinical information in patients with normal VLCFA testing, for which we typically did not have a referral reason. As described in other reports [6], most of our samples were reported out as normal, and no further follow-up with the provider was pursued. The published literature estimated a 15% false-negative rate in female heterozygotes for ALD with this test [6], which is less sensitive than measuring C26:0-LPC in dried blood spots or plasma [33,34]. We were not able to assess in our cohort the frequency of patients with an abnormal NBS that had normal VLCFA testing in plasma. Additionally, even for the diagnostic abnormal results, recontacting the ordering physician to further collect information or clarify the reason of referral was not always possible, and when information was obtained, it was occasionally incomplete. Furthermore, the clinical information relayed to us typically stemmed from the initial patient’s clinical assessment that had triggered VLCFA testing, often following the onset of neurologic symptoms, and may not yet reflect a full, multidisciplinary evaluation. We utilized all the available patient information (demographic data, phenotype description, family history) and laboratory test results to reach a high degree of confidence in our analysis; however, unfortunately a definite confirmation was only provided in about 40% of diagnostic results. Our study highlights the importance of providing patients’ information not only to improve result interpretation, but also to enrich patient datasets, potentially used for retrospective analysis to assess test utility.

Even with these limitations, our retrospective analysis showed an increase in abnormal results consistent with peroxisomal disorders after NBS inclusion, mostly reflecting patients identified because of a positive newborn screen, either in the proband or in a family member. In part, the increase in detection could be due to increased disease awareness following NBS implementation. We were also able to confirm a statistically significant difference in age at diagnosis and male-to-female ratio in patients with ALDP deficiency relative to the reason of referral. Most patients referred because of clinical findings were males, with 31% being 6 to 12-year-old boys, in contrast to most patients identified via NBS, who were less than a year of age and with almost half being females. Not surprisingly, in our study and in the literature, 6 to 12-year-old boys are also the most likely to present clinically with cerebral ALD. 

This study confirms that the implementation of NBS for ALD has allowed for the pre-symptomatic detection of ALDP deficiency, including of the severe cerebral form. Treatment of CCALD with hematopoietic stem cell transplantation has associated risks, but it is effective at halting progression of the disease if performed early [23]. Our data also showed that NBS has made a significant impact on the detection of female heterozygotes and of patients with peroxisome biogenesis defects of the Zellweger spectrum. Lacking an effective treatment, these are still challenging consequences of the program. However, identifying these patients early or, in some cases, asymptomatically, could significantly shorten the diagnostic process, have important implications for family planning, and increase the opportunity for participation in clinical trials.

## Figures and Tables

**Table 1:** Status of Adrenoleukodystrophy newborn screening by state, as of January 2023.

State	Included	Start Date	State	Included	Start Date
Alabama	No (*)		Montana	No	
Alaska	No		Nebraska	Yes	1 July 2018
Arizona	Yes	1 January 2022	Nevada	No	
Arkansas	Yes	15 November 2021	New Hampshire	Yes	26 August 2020
California [10]	Yes	(^a^) 21 September 2016	New Jersey	Yes	8 July 2019
Colorado	No(*)		New Mexico	Yes	1 January 2023
Connecticut	Yes	1 July 2016 ^a^	New York [9]	Yes	30 December 2013
Delaware	Yes	January 2020	North Carolina [12]	Yes	2 January 2018
District of Columbia	Yes	September 2018	North Dakota	No	
Florida	Yes	1 May 2018	Ohio	Yes	October 2022
Georgia [13]	Yes	May 2020	Oklahoma	Yes	March 2021
Hawaii	No		Oregon	Yes	1 January 2023
Idaho	Yes	1 February 2022	Pennsylvania [15]	Yes	April 2017
Illinois [14]	Yes	18 June 2019	Rhode Island	Yes	1 October 2018
Indiana	Yes	1 July 2021	South Carolina	No	
Iowa	No		South Dakota	No	
Kansas	No		Tennessee	Yes	30 April 2018
Kentucky	Yes	9 July 2018	Texas	Yes	5 August 2019
Louisiana	No		Utah	Yes	20 September 2020
Maine	Yes	1 April 2022	Vermont	Yes	1 May 2019
Maryland	No		Virginia	Yes	March 2022
Massachusetts	Yes	29 January 2018	Washington	Yes	1 March 2018
Michigan	Yes	October 2019	West Virginia	Yes	April 2020
Minnesota [11]	Yes	6 February 2017	Wisconsin	No	
Mississippi	No		Wyoming	No	
Missouri	Yes	1 December 2021			

(*) Expected soon. (^a^) Pilot program started in October 2015.

**Table 2:** ADLP deficiency phenotypes observed in the subset of patients with a diagnostic profile and detailed clinical information (279 out of 506 total number of diagnostic results).

Phenotype	ALDP Deficiency	
	Number of Patients (Males/Females)	Age in Years (Median; Range)
Asymptomatic	138 (80/58)	0.11; 0.01–73
Primary adrenal insufficiency (only)	23 (22/1)	9.3; 1.8–62
Myelopathy with or without peripheral neuropathy (adrenomyeloneuropathy, AMN)	80 (50/30)	46; 5.3–78
Rapidly progressive, inflammatory white matter demyelination (cerebral ALD)	38 (38/0)	8.9; 5.2–60

**Table 3:** Reasons for sample referral in patients with results diagnostic of ALDP deficiency compared to patients with results diagnostic of Zellweger spectrum disorders. Referral information was available in 504/614 cases.

Reason for Referral	ALDP Deficiency		PBD-ZSD	
	Number of Patients (Males/Females)	Age in Years (Median; Range)	Number of Patients (Males/Females)	Age in Years (Median; Range)
Clinical findings	179 (145/34)	21; 1.2–78	71 (40/31)	0.07; 0–50
Family history	58 (26/32)	23; 0.27–74	4 (2/2)	1.2; 0–4.4
Positive NBS in proband	137 (70/67)	0.07; 0.01–2.5	25 (12/13)	0.03; 0–0.42
Positive NBS in family member	30 (24/6)	15; 0.06–61	0	N/A

PBD-ZSD = peroxisome biogenesis defects-Zellweger spectrum disorders; N/A = not applicable.

## Data Availability

The data presented in this study may be available on request from the corresponding author. The data are not publicly available due to patient privacy.
